# The potential role of insulin resistance in predicting outcome from intravenous thrombolytic therapy

**DOI:** 10.1007/s13760-022-02060-6

**Published:** 2022-08-20

**Authors:** Mona Ali, Mona Hussein, Rehab Magdy, Ahmed Khamis, Asmaa M. Othman, Shaimaa A. Abdelkareem, Wesam Osama

**Affiliations:** 1 Department of Neurology, Beni-Suef University, Beni-Suef, Egypt; 2 Department of Neurology, Cairo University, Cairo, Egypt; 3 Beni-Suef General Hospital, Beni-Suef, Egypt; 4 Department of Internal Medicine, Beni-Suef University, Beni-Suef, Egypt; 5 Department of Clinical and Chemical Pathology, Beni-Suef University, Beni-Suef, Egypt

**Keywords:** Insulin resistance, Metabolic syndrome, HOMA-IR, Acute stroke, Rt-PA outcome

## Abstract

**Background:**

The potential impact of insulin resistance on stroke prognosis after IV thrombolysis is poorly understood. This study aimed to assess the effect of insulin resistance and metabolic syndrome on the outcome of IV thrombolysis in non-diabetic patients with acute ischaemic stroke.

**Methods:**

This prospective observational study was conducted on 70 non-diabetic acute ischaemic stroke patients who received rt-PA within 3 h of stroke onset. Patients were subjected to baseline and follow-up NIHSS measurements at 24 h and 3 months post-treatment. Stroke outcome was assessed after 3 months using the Modified Rankin Scale (mRS). The homeostasis model assessment–insulin resistance (HOMA-IR) was calculated for the included patients at stroke onset.

**Results:**

The mean age of included patients was 57.04 ± 14.39 years. Patients with unfavourable outcome had a significantly higher frequency of insulin resistance and metabolic syndrome, higher values of baseline NIHSS, insulin, HOMA-IR, uric acid and lower levels of HDL than those with favourable outcome (*P* value = 0.035, 0.007, ≤ 0.001, 0.001, ≤ 0.001, 0.002, 0.033, respectively). Each point increase in NIHSS before rt-PA increased the odds of an unfavourable outcome by 2.06 times (95% CI 1.22 − 3.478). Also, insulin resistance increased the odds of the unfavourable outcome by 11.046 times (95% CI 1.394–87.518). There was a statistically significant improvement in NIHSS 3 months after receiving rt-PA in all patients, significantly higher in patients who did not have insulin resistance or metabolic syndrome.

**Conclusion:**

Insulin resistance and metabolic syndrome were associated with worse functional outcomes in non-diabetic stroke patients after receiving rt-PA.

## Introduction

Stroke is the main leading cause of mortality and disability in adults [1]. Nowadays, intravenous thrombolysis with recombinant tissue plasminogen activator (rt-PA) is the primary reperfusion therapy with proven efficacy for managing acute ischaemic stroke, saving cerebral ischaemic tissue and reducing neurologic sequelae [2]. Unfortunately, the functional outcome after rt-PA thrombolysis is not always optimal, as some patients may still have some neurological deficits.

Insulin resistance (IR) is recognized as an impaired response of target tissues to insulin. Insulin resistance impairs glucose metabolism, leading to a compensatory increase in insulin production and hyperinsulinemia and may subsequently lead to metabolic syndrome [3].

Both insulin resistance and diabetes may lead to a prothrombotic and proinflammatory state through impairment of endogenous fibrinolysis and increased platelet activation, explaining their known association with a higher incidence of stroke [4].

Several tools exist for the quantitative assessment of insulin resistance. The homeostasis model assessment-insulin resistance (HOMA-IR) serves as a reliable method of estimating insulin resistance widely used in clinical studies [5].

While previous studies have confirmed the association between diabetes and poor functional outcome of stroke after thrombolysis [6, 7], studies regarding insulin resistance in this area are scarce [8, 9].

This study investigates the effect of insulin resistance and metabolic syndrome on the outcome of IV thrombolysis in non-diabetic patients with acute ischaemic stroke.

## Methods

This prospective observational study was carried out at the Stroke Unit of Beni-Suief University Hospital from January 2021 to December 2021. We included 70 patients presenting with acute ischaemic stroke who were in the therapeutic window for treatment with rt-PA (within the first 3 h) and had no contraindication for rt-PA injection according to guidelines set by The American Heart Association/American Stroke Association [10]. The included patients should be over ≥ 18 years.

**Fig. 1:**
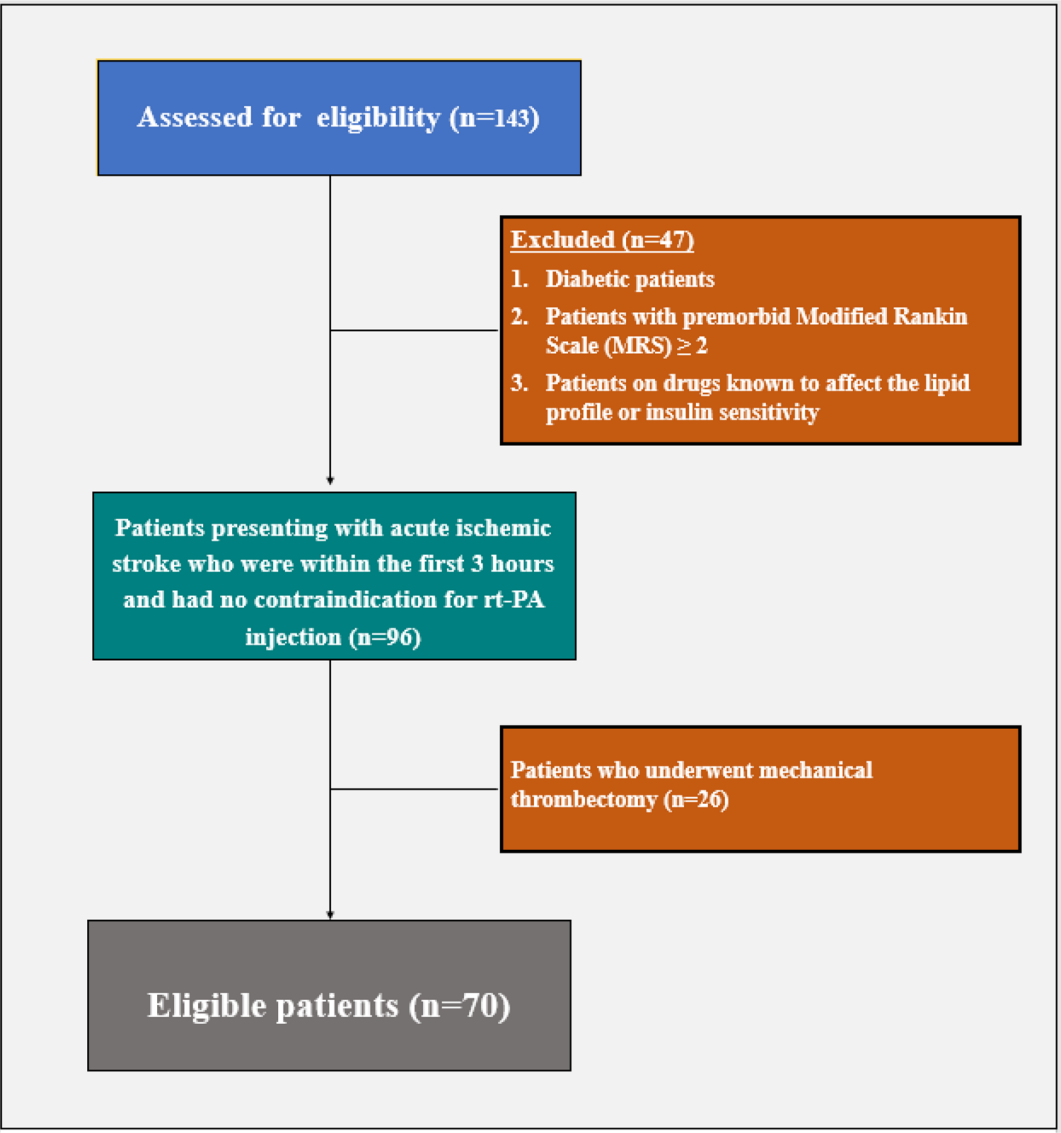
Flow diagram for the included and the excluded patients

Diabetic patients whose diagnosis was made by either fasting blood glucose (FBG) levels ≥ 126 mg/dL or 2-h plasma glucose ≥ 200 mg/dL or HbA1c ≥ 6.5% [11], patients with premorbid Modified Rankin Scale (MRS) ≥ 2, patients who underwent mechanical thrombectomy were excluded. Also, patients on drugs known to affect the lipid profile or insulin sensitivity were excluded (statins, fibrates, steroids, oral contraceptives, antipsychotics, B blockers, and thiazide diuretics). A flow diagram for the included and the excluded patients was illustrated in Fig. 1

### Study measures

Demographic information and stroke risk factors evident by history and supported by appropriate investigations were collected. Patients were coded with substance use disorder (SUD) if they were fulfilling the criteria of the Diagnostic and statistical manual of mental disorders (DSM-V) [12]. Body mass index (BMI) was calculated as weight divided by height squared (kg/m^2^). Waist circumference (to the nearest 0.5 cm) was measured using a plastic tape measure midway between the last rib and the iliac crest at the end of normal expiration.

The stroke subtype was determined according to the Org 10,172 trial in the acute stroke treatment (TOAST) categories [13]. The National Institute of Health Stroke Scale (NIHSS) [14] was recorded for the included patients at stroke onset and 24 h post-treatment.

Size of the infarction was measured by the pure ellipsoid model of ABC/2. It was found by Sims et al. to be the best model for a rapid and accurate clinical estimation of stroke volume [15]. In addition to the initial brain imaging, computerized tomography (CT) scan of the brain was performed 24 h following the treatment (and if there was any deterioration in NIHSS) to assess the occurrence of symptomatic intracerebral haemorrhage (sICH). In line with the Safe Implementation of Thrombolysis in Stroke-Monitoring Study (SITS-MOST) criteria [16], sICH is defined as a neurological deterioration of ≥ 4 points on the NIHSS from baseline within 24 h combined with haemorrhage on follow-up by neuroimaging.

According to the definition of metabolic syndrome provided by the National Cholesterol Education Program (NCEP) Adult Treatment Panel III (ATP III) report [17], three or more of the following were required to diagnose metabolic syndrome in the included patients: (1) FBG ≥ 100 mg/dL; (2) elevated blood pressure (≥ 130/80 mmHg) (3) HDL cholesterol of < 40 mg/dL for men and < 50 mg/dL for women; (4) triglycerides > 150 mg/dL, and (5) a waist circumference of > 90 cm for men and > 80 cm for women.

### Laboratory work-up

- High-density lipoprotein cholesterol (HDL-C) and Triglycerides (TGs) levels.
- Fasting glucose.
- Fasting insulin
- HOMA-IR was calculated based on the following equation: fasting insulin (μU/mL) × fasting glucose (mmol/L)/22.5 [18]. The state of insulin resistance was established if the HOMA-IR value > 2.5 [19].

Serum samples venous blood samples of 4 mL were withdrawn within 24 h from stroke onset. Samples were allowed to clot for 1 h at room temperature before centrifugation for 15 min at 3000×*g*. Serum was separated, and samples were divided. One part was sent for chemical analysis (triglycerides, total cholesterol, HDL), and 2nd part was stored at – 80 °C until the end of the study to measure serum fasting insulin by quantitative sandwich enzyme immunoassay technique ELISA kit Thermo Fisher.

### Study outcome

According to the hospital policy, patients were re-evaluated by NIHSS and the Modified Rankin Scale (mRS) score during a face-to-face interview 90 days following stroke. A favourable outcome at 90 days following stroke is defined as mRS scores of 0–2, whereas scores of 3–6 define the unfavourable outcome [20].

### Sample size calculation

The sample size was calculated using Epi calc 2000, version 1.02, 1997. Based on an alpha level of significance of 0.05, and 40.3% prevalence rate of metabolic syndrome in stroke patients with good outcome after IV thrombolysis and 88.7% prevalence rate in those with poor outcome after IV thrombolysis [21], a total sample size of at least 60 patients was required to achieve a statistical power of 99%.

### Ethical statement

Written informed consent was obtained from each patient or their first-degree relatives. The study was performed in accordance with the Declaration of Helsinki. The study was approved by the Ethics Committees of the Beni-Suef University; the approval number was FWA00015574.

### Statistical analysis

IBM SPSS (Statistical Package of Social Science) Version 25 was used to analyze the data. Categorical variables were expressed as numbers and percentages. Normally distributed quantitative variables were expressed as mean and standard deviation (SD), while non-normally distributed variables were expressed as the median and interquartile range (IQR). Chi-squared test was used for comparison between patients with favourable and unfavourable outcome in categorical variables. The Independent sample *t* test was used to compare the two groups in normally distributed quantitative variables. In contrast, the Mann–Whitney test was used to compare them in non-normally distributed quantitative variables. A mixed ANOVA test was used to compare NIHSS before and 3 months after thrombolytic therapy in patients with and without metabolic syndrome and in those with and without insulin resistance. A binary logistic regression model was done to identify predictors of poor outcome from thrombolytic therapy after being adjusted for their potential mutual confounding effect. *P* value ≤ 0.05 was considered statistically significant. All tests were two-tailed.

## Results

### Demographics, clinical and laboratory characteristics of the included patients

This prospective observational study was conducted on 70 acute ischaemic stroke patients eligible for rt-PA treatment (45 males and 25 females). The mean age of the included patients was 57.04 ± 14.39 years. The mean BMI and waist circumference values in cm were 29.75 ± 4.66 and 94.67 ± 12.48, respectively. Regarding stroke risk factors, 72.9% of the included patients were hypertensive, 47.1% had AF, 44.3% were smokers, 8.6% were drug abusers, and 25.7% had a family history of stroke.

Regarding stroke subtype, cardio-embolic stroke represented the most frequent subtype that was reported in 45.7% of the whole study population, followed by small artery disease (34.3%), large artery disease (12.9%), other determined (4.3%), and lastly undetermined (2.9%).

The median value for door-to-needle time (DTN) was 32.5 min with IQR (30–45). The mean value for NIHSS before rt-PA was 13.61 ± 4.44; after 24 h was 11.24 ± 5.46; and after 3 months was 6.11 ± 4.83. The median value for infarction size was 9.365 cm^3^ with IQR (4.22–19.57).

After receiving rt-PA, eight patients (11.4%) experienced complications (5 patients had reperfusion injury and three patients had haemorrhagic transformation). At the 3-month follow-up, 47.1% of the included patients had a favourable outcome, while 52.9% had an unfavourable outcome. Forty percent of patients had insulin resistance, and 57.1% had metabolic syndrome (Table 1).

**Table 1:** Demographics, clinical and laboratory characteristics of the included patients

	Patients (*n* = 70)
Age in years [mean (SD)]	57.04 (14.39)
Sex	
Males [*n* (%)]	45 (64.3%)
Females [*n* (%)]	25 (35.7%)
BMI [mean (SD)]	29.75 (4.66)
Waist circumference in cm [mean (SD)]	94.67 (12.48)
HTN [*n* (%)]	51 (72.9%)
AF [*n* (%)]	33 (47.1%)
Smoking [*n* (%)]	31 (44.3%)
Substance use disorder [*n* (%)]	6 (8.6%)
Family history of stroke [*n* (%)]	18 (25.7%)
Stroke subtype by TOAST classification	
Small vessel occlusion [*n* (%)]	24 (34.3%)
Large artery atherosclerosis [*n* (%)]	9 (12.9%)
Cardio-embolic [*n* (%)]	32 (45.7%)
Other determined [*n* (%)]	3 (4.3%)
Undetermined [*n* (%)]	2 (2.9%)
DTN in minutes [median (IQR)]	32.5 (30–45)
NIHSS	
Before rt-PA [mean (SD)]	13.61 (4.44)
After 24 h [mean (SD)]	11.24 (5.46)
After 3 months [mean (SD)]	6.11 (4.83)
Complications from rt-PA [*n* (%)]	8 (11.4%)
mRS after 3 months	
Favourable [*n* (%)]	33 (47.1%)
Unfavourable [*n* (%)]	37 (52.9%)
Infarction size in cm^3^ [median (IQR)]	9.365 (4.22–19.57)
Laboratory work up	
HDL [mean (SD)]	41.16 (10.53)
TGs [mean (SD)]	138.81 (68.19)
FBS [mean (SD)]	97.3 (17.71)
Insulin [mean (SD)]	9.26 (4.89)
HOMA-IR [mean (SD)]	2.29 (1.29)
Uric acid [mean (SD)]	6.9 (1.96)
Insulin resistance [*n* (%)]	28 (40%)
Metabolic syndrome [*n* (%)]	40 (57.1%)

*AF* Atrial fibrillation, *BMI* Body mass index, *DTN* Door to needle, *FBS* Fasting blood sugar, *HDL* High-density lipoprotein, *HOMA-IR* Homeostatic Model Assessment-insulin resistance, *HTN* Hypertension, *mRS* Modified Rankin scale, *NIHSS* National Institutes of Health Stroke Scale, *rt-PA* Recombinant tissue plasminogen activator, *TGs* Triglycerides, *TOSAT* Trial of Org 10,172 in Acute Stroke Treatment

### Outcome from thrombolytic therapy (assessed by mRS)

The group of patients with unfavourable outcome (*n* = 37) had a significantly higher frequency of males and smokers, higher mean values of baseline NIHSS, FBS, insulin, HOMA, uric acid, and lower mean values of HDL than the group of patients with favourable outcome (*n* = 33) (*P* value = 0.035, 0.007, ≤ 0.001, ≤ 0.001, 0.001, ≤ 0.001, 0.002, 0.033, respectively). The frequency of both insulin resistance and metabolic syndrome was significantly higher in patients with unfavourable outcomes than in those with favourable outcomes (*P *value ≤ 0.001, 0.019, respectively) (Table 2).

**Table 2:** Factors affecting outcome from thrombolytic therapy (assessed by mRS)

	Patients with favourable outcome (*n* = 33)	Patients with unfavourable outcome (*n* = 37)	*P* value
Age in years [mean (SD)]	53.58 (15.16)	60.14 (13.11)	0.056
Sex			
Males [*n* (%)]	17 (51.5%)	28 (75.7%)	0.035*
Females [*n* (%)]	16 (48.5%)	9 (24.3%)	
BMI [mean (SD)]	29.03 (4.6)	30.4 (4.67)	0.223
Waist circumference in cm [mean (SD)]	94.61 (12.27)	94.73 (12.84)	0.967
HTN [*n* (%)]	21 (63.6%)	30 (81.1%)	0.101
AF [*n* (%)]	13 (39.4%)	20 (54.1%)	0.220
Smoking [*n* (%)]	9 (27.3%)	22 (59.5%)	0.007*
Substance use disorder [*n* (%)]	2 (6.1%)	4 (10.8%)	0.479
TOAST classification			
Small artery [*n* (%)]	10 (30.3%)	14 (37.8%)	0.076
Large artery [*n* (%)]	6 (18.2%)	3 (8.1%)	
Cardio-embolic [*n* (%)]	12 (36.4%)	20 (54.1%)	
Other determined [*n* (%)]	3 (9.1%)	0 (0%)	
Undetermined [*n* (%)]	2 (6.1%)	0 (0%)	
DTN [median (IQR)]	30 (27.5–45)	35 (30–47.5)	0.741
NIHSS Before rt-PA [mean (SD)]	10.67 (2.88)	16.24 (3.91)	≤ 0.001*
Infarction size in cm^3^ [median (IQR)]	9.08 (3.59–19.86)	9.65 (4.77–19.38)	0.962
Laboratory work up			
HDL [mean (SD)]	43.98 (9.76)	38.65 (10.69)	0.033*
TGs [mean (SD)]	131.42 (74.56)	145.41 (62.27)	0.396
FBS [mean (SD)]	89.45 (16.38)	104.3 (15.99)	≤ 0.001*
Insulin [mean (SD)]	7.19 (4.6)	11.11 (4.43)	0.001*
HOMA-IR [mean (SD)]	1.66 (1.2)	2.84 (1.11)	≤ 0.001*
Uric acid [mean (SD)]	6.08 (1.8)	7.62 (1.82)	0.002*
Insulin resistance [*n* (%)]	6 (18.2%)	22 (59.5%)	≤ 0.001*
Metabolic syndrome [*n* (%)]	14 (42.4%)	26 (70.3%)	0.019*

*AF* Atrial fibrillation, *BMI* Body mass index, *DTN* Door to needle, *FBS* Fasting blood sugar, *HDL* High-density lipoprotein, *HOMA-IR* Homeostatic Model Assessment-insulin resistance, *HTN* Hypertension, *mRS* Modified Rankin scale, *NIHSS* National Institutes of Health Stroke Scale, *rt-PA* Recombinant tissue plasminogen activator, *TGs* Triglycerides, *TOSAT* Trial of Org 10,172 in Acute Stroke Treatment

*P value ≤ 0.05 is considered significant

A binary logistic regression model was done to identify the predictors of unfavourable outcome from thrombolytic therapy. Sex, smoking, NIHSS before rt-PA, HDL, uric acid, insulin resistance and metabolic syndrome were used as the independent variables.

Each point increase in NIHSS before rt-PA increased the odds of an unfavourable outcome by 2.06 times (95% CI 1.22 − 3.478). Also, insulin resistance increased the odds of the unfavourable outcome by 11.046 times (95% CI 1.394–87.518) (Table 3).

**Table 3:** Predictors of poor outcome from thrombolytic therapy (assessed by mRS)

	*B*	*P* value	Odds ratio	95% CI	
				Lower	Upper
Male sex	1.123	0.520	3.074	0.100	94.317
Smoking	0.801	0.607	2.228	0.105	47.269
NIHSS before rt-PA	0.723	0.007*	2.060	1.220	3.478
HDL	− 0.062	0.440	0.940	0.802	1.100
Uric acid	0.174	0.539	1.190	0.683	2.073
Insulin resistance	2.402	0.023*	11.046	1.394	87.518
Metabolic syndrome	0.713	0.594	2.040	0.148	28.099
Constant	− 10.600	0.060	0.000		

*HDL* high-density lipoprotein, *NIHSS* National Institutes of Health Stroke Scale, *rt-PA* Recombinant tissue plasminogen activator

Nagelkerke *R* Square = 0.792, *P value ≤ 0.05 is considered significant

### Impact of insulin resistance and metabolic syndrome on the outcome from thrombolytic therapy (assessed by NIHSS)

There was a statistically significant improvement in the scores of NIHSS 3 months after receiving rt-PA in all included patients, whether they had insulin resistance or metabolic syndrome or not (*P* value ≤ 0.001 in all parameters). Such improvement was significantly higher in patients who didn’t have insulin resistance or metabolic syndrome (*P* value = 0.046, 0.005, respectively) (Table 4).

**Table 4:** Effect of insulin resistance and metabolic syndrome on the outcome from thrombolytic therapy (assessed by NIHSS)

	NIHSS before rt-PA [mean (SD)]	NIHSS after 3 months [mean (SD)]	*P* value	*P* value between groups
Insulin resistance				
Yes (*n* = 28)	14.86 (4.31)	7.46 (4.17)	≤ 0.001*	0.046*
No (*n* = 42)	12.79 (4.38)	5.21 (5.07)	≤ 0.001*	
Metabolic syndrome				
Yes (*n* = 40)	14.8 (4.77)	7.48 (5.17)	≤ 0.001*	0.005*
No (*n* = 30)	12.03 (3.43)	4.3 (3.69)	≤ 0.001*	

*NIHSS* National Institutes of Health Stroke Scale, *rt-PA* Recombinant tissue plasminogen activator

*P value ≤ 0.05 is considered significant

### Impact of insulin resistance and metabolic syndrome on the occurrence of complications from thrombolytic therapy

Patients with metabolic syndrome had a significantly higher incidence of complications from rt-PA than those without (*P* value = 0.009). In contrast, there was no statistically significant difference between patients with and without insulin resistance regarding the incidence of complications (*P* value = 0.878) (Table 5).

**Table 5:** Effect of insulin resistance and metabolic syndrome on the occurrence of complications from thrombolytic therapy

	Patients with complications (*n* = 8)	Patients without complications (*n* = 62)	*P *value
Insulin resistance			
Yes [*n* (%)]	3 (37.5%)	25 (40.3%)	0.878
No [*n* (%)]	5 (62.5%)	37 (59.7%)	
Metabolic syndrome			
Yes [*n* (%)]	8 (100%)	32 (51.6%)	0.009*
No [*n* (%)]	0 (0%)	30 (48.4%)	

*P value ≤ 0.05 is considered significant

## Discussion

It has long been recognized that hyperglycaemia in acute ischaemic stroke patients is associated with unfavourable functional outcomes [22]. Therefore, stroke treatment guidelines [10] recommend the normalization of blood glucose levels. Yet, no recommendation has been made regarding insulin resistance.

This study emphasizes the negative effect of insulin resistance on functional outcome and dependency in stroke patients who received rt-PA (OR 11.046, 95% CI 1.394–87.518). Many studies showed significantly more favourable outcomes in patients with acute ischaemic stroke receiving rt-PA within 3 h than between 3 and 4.5 h of symptoms onset [23, 24]. For the purpose of eliminating possible confounding factors as much as possible, we excluded patients with acute ischaemic stroke who received rt-PA in the extended window (3–4.5 h).

Insulin resistance may promote various conditions that could worsen the response to intravenous thrombolysis and stroke outcome, such as defective endogenous fibrinolysis, increased platelet activation, endothelial dysfunction, and a chronic proinflammatory state [25]. Moreover, impaired synaptic plasticity, excessive reactive oxygen species and mitochondrial dysfunction mediated by insulin resistance may interfere with functional recovery from the stroke insult [26].

Similar work conducted by Yang, Li [9] showed that insulin resistance was significantly associated with a high probability of haemorrhagic transformation as well as a poor functional outcome at 90 days in non-diabetic ischaemic stroke patients treated with intravenous thrombolysis. Yet, the authors did not investigate the effect of metabolic syndrome or its components on the stroke outcome.

The current study found that improvement of NIHSS 3 months after receiving rt-PA was significantly higher in patients without metabolic syndrome than in those with metabolic syndrome. Furthermore, patients with metabolic syndrome had a significantly higher frequency of complications from rt-PA than those without metabolic syndrome. It is well established that the state of metabolic syndrome is characterized by defective endogenous fibrinolysis with an enhancement of fibrinolysis inhibitors like plasminogen activator inhibitor-1 (PAI-1) [27], which may account for decreased efficacy of clot lysis after rt-PA administration. It should be noted that metabolic syndrome represents a fundamental risk factor not only for atherosclerosis but also for cardioembolic strokes. It has been found that chronic hypertension, dyslipidaemia and elevated leptin levels promote atrial fibrosis and angiotensin II-evoked AF [28].

On studying the other components of metabolic syndrome individually in relation to poor functional outcome, the only factor significantly associated with the unfavourable outcome was lower mean values of HDL. This can be explained by the fact that HDL has well-known action of reverse cholesterol transport in which HDL transports cholesterol from the arterial wall to the liver for excretion. Moreover, it has an anti-inflammatory, antioxidant and endothelial protective action. HDL may also reduce the thrombotic risk by inhibiting platelet functions and improving endothelial function by stimulating the release of the prostacyclin [29, 30].

On the other hand, other components of the metabolic syndrome, including serum triglycerides, hypertension, and waist circumference, were not significantly associated with the unfavourable outcome after intravenous thrombolysis, contrary to the previous studies [31–33].

Another significant independent predictor of unfavourable stroke outcome found in this study that has to be mentioned is higher baseline NIHSS, which is in line with previous studies [34–37]. The current study showed that each point increase in NIHSS before rt-PA administration nearly doubles the odds of an unfavourable outcome (OR 2.06, 95% CI 1.22 − 3.478).

Finally, it should be noted that the main limitation of our study is that we did not investigate the effect of insulin resistance and metabolic syndrome in relation to symptomatic cerebral haemorrhage after treatment with rt-PA due to the limited number of patients who developed this complication.

## Conclusion

Insulin resistance and metabolic syndrome were associated with worse functional outcomes in non-diabetic stroke patients after receiving IV thrombolysis. These findings may strengthen the importance of screening and proper management of insulin resistance and metabolic syndrome in patients at risk of acute ischaemic stroke.

## Data Availability

Authors report that the datasets used and/or analyzed during the current study are available from the corresponding author on reasonable request.
